# The presence and distribution of various genes in postnatal CLP-affected palatine tissue

**DOI:** 10.1186/s40902-024-00412-1

**Published:** 2024-01-16

**Authors:** Jana Goida, Mara Pilmane

**Affiliations:** Institute of Anatomy and Anthropology, Riga Stradins University, Riga, LV-1010 Latvia

**Keywords:** Cleft lip and palate, PAX7, PAX9, SHH, SOX3, WNT3A, WNT9B

## Abstract

**Background:**

Worldwide cleft lip with or without a cleft palate (CL/P) is the most common craniofacial birth defect. Apart from changes in facial appearance, additionally affected individuals often suffer from various associated comorbidities requiring complex multidisciplinary treatment with overall high expenses. Understanding the complete pathogenetic mechanisms of CL/P might aid in developing new preventative strategies and therapeutic approaches, help with genetic counselling, and improve quality of life. Many genes have been associated with the development of orofacial clefts; however, the majority require further research. Based on the role of PAX7, PAX9, SHH, SOX3, WNT3A, and WNT9B in orofacial development, the intention was to use chromogenic in situ hybridization to detect the six genes in postnatal CLP-affected palatine tissue and compare their distribution within the tissue samples.

**Results:**

Statistically significant differences in the distribution of PAX7, PAX9, WNT3A, and WNT9B were observed. In total, 19 pairs of moderate to very strong positive correlations were noted.

**Conclusions:**

Changes in the cleft-affected palatine epithelium primarily seem to be associated with the PAX7 gene; however, PAX9, WNT3A, WNT9B, and SOX3 role seems to be more limited. Whilst connective tissue changes seem to depend on PAX7 only, SHH seems to participate individually and indistinctly. Numerous positive correlations reflect the complicating interactions of the pathways and their components in the orofacial cleft morphopathogenesis.

**Supplementary Information:**

The online version contains supplementary material available at 10.1186/s40902-024-00412-1.

## Background

Orofacial clefts (OFC) are among the most common birth defects worldwide. The approximate prevalence in European populations is 1/1000 live births [1]. Overall, the majority (~ 70%) of orofacial clefts are thought to be non-syndromic [2]. Depending on the defect, orofacial clefts are divided into unilateral or bilateral, complete or incomplete, and may involve cleft lip only (CLO), isolated cleft palate (CPO), or cleft lip with cleft palate (CLP) [3]. Unfortunately, apart from aesthetical defects, typically patients present with other associated comorbidities, for instance, feeding difficulties, speech problems, dental malocclusion, dysgnathia of varying degrees, middle ear infection, anemia, respiratory and cardiovascular diseases [4, 5]. Furthermore, studies have reported a negative impact on the quality of life of the patients and their families, with social-emotional wellness being one of the most affected domains [6–8]. Therefore, team-based, interdisciplinary care, which involves, e.g., surgical, dental, speech, and psychological services, and allows to provide necessary treatment at the appropriate age and in the right order, is essential. Consequently, greater healthcare costs among children with orofacial clefts have been reported [9].

Non-syndromic OFCs are thought to be multifactorial and due to their etiopathological complexity, attempts to identify the causal associations have not been as successful as with syndromic anomalies [2]. Currently, more than 450 potential CL/P genes have been reported in mice and humans; however, the majority require further research [10].

PAX7 and PAX9 genes are a part of the paired box (PAX) gene family, which encodes nuclear transcriptional factors and, by tightly regulating the expression of genes in a cell-type specific manner, ensures correct cell lineage throughout development and maintains cell identity in adulthood [11, 12]. During development, the expression of PAX7 and PAX9 is diverse [12]. PAX7 has been observed in the central nervous system (CNS), neural crest, and muscles, but PAX9 in the sclerotome, skeleton, craniofacial tissue, teeth, and thymus [11]. It has been hypothesized that PAX7 plays a key role in the integration of inputs during neural crest induction and in controlling the specification of neural crest derivates, however, PAX9 seems to participate in epithelial-mesenchymal communications of palate growth, palatal shelf elevation, and fusion [13, 14]. Pax7 and Pax9 have been linked to orofacial clefts in mice models [13–18] and different human studies [19–23].

Sonic hedgehog (SHH), a member of the hedgehog family, secretes proteins that act upon intracellular and distantly located cells to mediate relevant gene expression and is involved in the development of the CNS, axial bone, limbs, teeth, et cetera [24]. During development, mutations, deficiencies, and abnormal expression of hedgehog proteins result in malformations, hyperplasia, and growth retardation in tissues, particularly in the skeleton, craniofacial structures, and CNS [25]. Overall, the SHH pathway is an important early regulator in reciprocal epithelial-mesenchymal communications controlling epithelial and mesenchymal proliferation [14]. Studies have shown that temporally-specific inhibition of Shh signaling in mice models has led to orofacial cleft formation [26, 27]. Furthermore, in human studies, different SHH etiological susceptibility variants have been reported [28, 29]. Additionally, statistically significant differences in SHH protein expression between the control group and OFC patients have been observed in immunohistochemically stained and examined tissue samples [30].

SRY-related HMG-box gene 3 (SOX3) is an X-linked member of the SOX gene family of transcriptional factors, and together with SOX1 and SOX2 comprises the SOXB1 subgroup [31, 32]. It is necessary for the formation of the hypothalamic-pituitary axis, and its expression is detected throughout the developing CNS [33]. Sox3 deletion in mice has resulted in pleiotropic phenotypes, including craniofacial development defects, abnormal tooth development, CNS defects, hypopituitarism, and affected spermatogenesis [34–36]. Similarly to mice, SOX3 mutations in humans also cause a variable phenotype [37–39]. Furthermore, increased SOX3-positive immunoreactive structures within human unilateral cleft lip have been reported [30].

WNT3A and WNT9B are members of the WNT (Wingless-type MMTV integration site) family, which secret glycolipoproteins. WNT signaling is essential for directing cell proliferation and polarity, as well as cell fate determination during development and tissue homeostasis [40]. During craniofacial development, Wnt signaling has been reported in most tissues [41]. Furthermore, the results of both mice and human studies have revealed a strong association between OFC and WNT3A, WNT9B [42–47]. WNT3A is thought to be involved in neural crest cell differentiation and migration and regulation of the epithelial-mesenchymal transition, which is required for normal palatal formation [47]. However, Wnt9b deletion in mice results in retarded outgrowth of the nasal and maxillary processes possibly due to reduced proliferation of mesenchymal cells, which leads to failure of physical contact between the facial processes [42].

The available literature strongly indicates PAX7, PAX9, SHH, SOX3, WNT3A, and WNT9B to be strong candidate genes for cleft lip with or without cleft palate. Moreover, it is understood that orofacial clefts together with their comorbidities cause a substantial burden on the patients and their families. Thus, identifying causal associations and morphopathogenetic mechanisms is relevant and significant to improve preventative strategies, treatment, and genetic counseling. There has been limited morphological research regarding the comparison of these genes within orofacial cleft tissue; ergo, taking into account the significance of these genes in craniofacial development, the objectives of this study were to examine the presence, distribution, and potential correlation between PAX7, PAX9, SHH, SOX3, WNT3A, and WNT9B in postnatal CLP-affected cleft palatine tissue.

## Methods

### Characterization of the study group

The research protocol was approved by the Ethics Committee of the Riga Stradins University, Latvia (Nr.6–1/10/11, 24.09.2020.; 2-PEK-4/492/2022, 21.11.2022.), and written informed consent was obtained from all the patients’ parents after a detailed explanation of the research.

The research group comprised 15 children (5 females, 10 males) diagnosed with Cheilognathouranoschisis dextra/sinistra/bilateralis. The inclusion criteria of the study group were: diagnosis of unilateral or bilateral CLP, no oral diseases or any other pathology hindering the patient from receiving repair of the palate. At the Institute of Stomatology, Riga Stradins University, Latvia, the patients’ palatine tissue samples were obtained during the surgical procedure by the same surgeon. No additional notes were written down by the doctor in 11 out of 15 cases. During pregnancy, paracetamol had been used in 2 cases. One case had a Down syndrome in family history, but another had cleft lip and palate. In detail, the main characteristics of the study group are summarized in Table S1, which can be viewed in the Supplementary file.

For the control group, five palatine tissue samples (2 females, 3 males), aged 2–3 years old, without any craniofacial pathologies were obtained from the archives at the Institute of Anatomy and Anthropology, Riga, Latvia. Two children died due to traumatic injuries; however, the cause of death of the remaining 3 was sudden unexplained death in childhood (SUDC).

### Chromogenic in situ hybridization

Chromogenic in situ hybridization (CISH) is a method which uses peptide nucleic acid probes to identify specific DNA/RNA sequences [48].

Immediately after veloplasty, palatine tissue samples were collected and fixed for 24 h in a mixture of formaldehyde (2%) and picric acid (0.2%) in 0.1M phosphate buffer (pH 7.2). Subsequently, the samples were rinsed in Tyrode’s buffer (content: NaCl, KCl, CaCl_2_·2H_2_O, MgCl_2_·6H_2_O, NaHCO_3_, NaH_2_PO_4_·H_2_O, glucose), which contained saccharose (10%), for 12 h and afterward embedded into paraffin. Tissue samples were registered and given codes.

Using ZytoDot2C CISH Implementation Kit (ZytoVision GmbH, Bremerhaven, Germany) with PAX7, PAX9, SHH, SOX3, WNT3A, and WNT9B probes (Empire Genomics Corp., Williamsville, NY, USA) CISH was performed. Following pretreatment, which was done using standard laboratory methods, the slides were denatured and hybridized. Initially, 10μL of the probe was added to the slide, and a clean 22 mm^2^ coverslip, avoiding trapped air bubbles, was applied to the slide. Afterwards, for 5 min the slides were placed on a 79 °C plate, then transferred to a humidity chamber and at 37 °C hybridized for 24 h. The following reagents are a part of the ZytoDot2C CISH Implementation Kit. The subsequent day, the coverslips were removed, and the specimens were immersed in Wash Buffer SSC for 5 min at 80 °C. Then twice rinsed with distillate water and immersed in Wash Buffer TBS. Afterwards, 1–2 drops of Anti-DIG/DNP-Mix were added to each slide and incubated in a humidity chamber for 15 min, 37 °C. Subsequently, once more immersed in newly made Wash Buffer TBS 3 times for the duration of 1 min. An AP-Red solution was prepared (1ml AP-Red Solution B + 1 drop (30 μL) AP-Red Solution A), 1–2 drops were added to each slide and left for 10 min at room temperature. Whilst waiting, an HRP-Green Solution (1 ml HRP-Green Solution B + 2 drops (2 × 20 μL) HRP-Green Solution A) was prepared. After 10 min, the specimens were rinsed for 2 min with distillate water. Afterward, 1–2 drops of HRP-Green Solution were added, and the slides were left for 10 min at room temperature. Then rinsed for 2 min with distillate water and colored with Nuclear Blue Solution for 2 min. The slides were moved into a staining jar, washed under cold running water for 2 min, dehydrated with 100% ethanol, and then incubated in xylene. Lastly, coverslips were reattached and the probe signals were visualized under a brightfield microscope. If the normal cells or cells without aberrations were in the interphase or metaphase, 2 brown-colored dots were anticipated to appear per nuclei.

The tissue samples were analyzed using a semi-quantitative scoring system (Table 1) by two independent researchers [49, 50]. Using immersion oil, the specimens were analyzed by evaluating the appearance of brown-colored dots in five or more randomly selected visual fields at × 1000 magnification. The presence of the brown dots was examined in 3 locations of the palatine mucosa—the epithelium, connective tissue, and endothelium.

**Table 1:** The semi-quantitative scoring system’s values and their description

Value	Description
0	No gene signals detected (0%) in the visual field
0/ +	Occasional gene signals detected (12.5%) in the visual field
+	Few gene signals detected (25%) in the visual field
+ / + +	Few to moderate gene signals detected (37.5%) in the visual field
+ +	Moderate number of gene signals detected (50%) in the visual field
+ + / + + +	Moderate to numerous gene signals detected (62.5%) in the visual field
+ + +	Numerous gene signals detected (75%) in the visual field
+ + + / + + + +	Numerous to abundant gene signals detected (87.5%) in the visual field
+ + + +	Abundance of gene signals detected (100%) in the visual field

Visualization of the tissue samples was performed using a Leica Leitz DM RB microscope (Leica Microsystems GmbH, Wetzlar, Germany) and Euromex DC.20000i camera (Euromex Microscopen bv, Arnhem, The Netherlands), but the image processing and analysis using ImageFocusAlpha software (Euromex Microscopen bv, Arnhem, The Netherlands).

### Statistical analysis

The data analysis was performed using IBM SPSS Statistics 27 (IBM Company, Armonk, New York, NY, USA). The semi-quantitative evaluation values were changed to numeric form, for instance, 0 to 0; 0/ + to 0.5; + to 1; etc. The Mann–Whitney* U* test was used to compare the distribution of PAX7, PAX9, SHH, SOX3, WNT3A, and WNT9B between the study and control groups. For the correlation analysis, Spearman’s rank correlation coefficient was applied. Interpretation of the *R* value was as follows: *R* + / − 0.00–0.19, a very weak correlation; *R* + / − 0.20–0.39, a weak correlation; *R* + / − 0.40–0.59, a moderate correlation; *R* + / − 0.60–0.79, a strong correlation; *R* + / − 0.80–1.0, a very strong correlation. In all statistical analyses, the statistical significance was determined by *p* value < 0.05.

## Results

In the majority of cases, gene-signal-containing cells were observed, with the exception of 2 patients (Patients No. 9 and No. 11), where no gene signals of PAX7, PAX9, SHH, SOX3, WNT3A, and WNT9B were observed in all three locations (Tables 2 and 3). Overall, the epithelium had the most gene-signal-containing cells, followed by the connective tissue and then the endothelium.

**Table 2:** Semi-quantitative evaluation of PAX7, PAX9, and SHH gene signals in cleft-affected palatine mucosa and controls

Patient’s No	PAX7			PAX9			SHH		
	E	CT	END	E	CT	END	E	CT	END
1	+ / + +	0	0	0/ +	0	0	0	0	0
2	0/ +	0	0	0	0	0	0	0	0
3	+ + / + + +	0/ +	0	0/ +	0	0	+ / + +	0	0
4	+ + + / + + + +	+ +	+	+ + / + + +	0	0	0	0	0
5	+ + +	+	0	+ + / + + +	0	0	0/ +	0	0
6	+ + + / + + + +	+ + / + + +	+ / + +	+ +	0	0	0/ +	0	0
7	+ / + +	0/ +	0	0/ +	0	0	0	0	0
8	+ + + / + + + +	+ + / + + +	+ / + +	+ + / + + +	0	0	+ / + +	0	0
9	0	0	0	0	0	0	0	0	0
10	+ + + / + + + +	0/ +	0	+ + / + + +	0	0	+ + / + + +	0	0
11	0	0	0	0	0	0	0	0	0
12	+ + +	0/ +	0	+ +	0	0	0	0	0
13	+ + / + + +	0	0	0	0	0	0/ +	0	0
14	+ + + / + + + +	+ / + +	0	+ + / + + +	0	0	0	0	0
15	0	0	0	0	0	0	0	0	0
Median	2.5	0.5	0.0	0.5	0.0	0.0	0.0	0.0	0.0
Q1–Q3	0.5–3.5	0.0–1.5	0.0–0.0	0.0–2.5	0.0–0.0	0.0–0.0	0.0–0.5	0.0–0.0	0.0–0.0
Control	0	0	0	0	0	0	0	0	0

*Abbreviations*: *No.* number, *E* epithelium, *CT* connective tissue, *END* endothelium

**Table 3:** Semi-quantitative evaluation of SOX3, WNT3A, and WNT9B gene signals in cleft-affected palatine mucosa and controls

Patient’s No	SOX3			WNT3A			WNT9B		
	E	CT	END	E	CT	END	E	CT	END
1	0	0	0	0/ +	0	0	0	0	0
2	0	0	0	0/ +	0	0	0/ +	0	0
3	+	0	0	+	0	0	+	0	0
4	+ +	0	0	+ +	0	0	+ + / + + +	0	0
5	+ +	0	0	0/ +	0	0	+ + + / + + + +	0	0
6	+ +	0	0	0/ +	0	0	+ + +	0	0
7	0	0	0	0/ +	0	0	0/ +	0	0
8	+ / + +	0	0	+ +	0	0	+ + +	0	0
9	0	0	0	0	0	0	0	0	0
10	+	0	0	+ + + / + + + +	0	0	+ / + +	0	0
11	0	0	0	0	0	0	0	0	0
12	+ + +	+	+ +	+ + + / + + + +	0	0	0	0	0
13	0	0	0	0/ +	0	0	0/ +	0	0
14	0/ +	0	0	+	0	0	+ + +	0	0
15	0/ +	0	0	0	0	0	0	0	0
Median	0.5	0.0	0.0	0.5	0.0	0.0	0.5	0.0	0.0
Q1–Q3	0.0–2.0	0.0–0.0	0.0–0.0	0.5–2.0	0.0–0.0	0.0–0.0	0.0–3.0	0.0–0.0	0.0–0.0
Control	0	0	0	0	0	0	0	0	0

*Abbreviations*: *No.* number, *E* epithelium, *CT* connective tissue, *END* endothelium

**Fig. 1:**
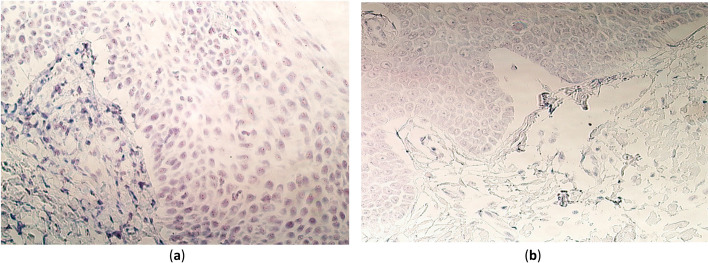
PAX7 gene visualization in cleft-affected palatine tissue of patient and control subjects using chromogenic in situ hybridization method and immersion oil. **a** A tissue sample of a 15-month-old child with numerous to abundant (+ +  + / +  +  + +) gene-signal-containing cells in the epithelium and a moderate number to numerous (+ + / +  + +) gene signals in the connective tissue. CISH, × 400. **b** Control tissue sample without any gene signals in the epithelial cells, connective tissue or endotheliocytes. CISH, × 400

No gene signals were detected in any of the 5 control tissue samples that were included in this study; ergo, the data were summarized in the form of a median and included in Tables 2 and 3. The control tissue samples are depicted in Fig. 1b and Figures S1a–e in the Supplementary file.

The presence of PAX7 gene signals within palatine epithelium varied greatly from no (0) to numerous to abundant (+ +  + / +  +  + +) signals detected. In fact, the median value of 2.5 (Q1–Q3, 0.5–3.5) is the largest in this study group (Table 2, Fig. 1a). Although the results in the connective tissue varied greatly, the range was narrower (from no gene signals (0) to moderate to numerous signals detected (+ + / +  + +)), and more tissue samples had 0 signals compared to the epithelium (Table 2). However, the majority of tissue samples (12 out of 15) had no PAX7 gene signals in the endothelium.

**Fig. 2:**
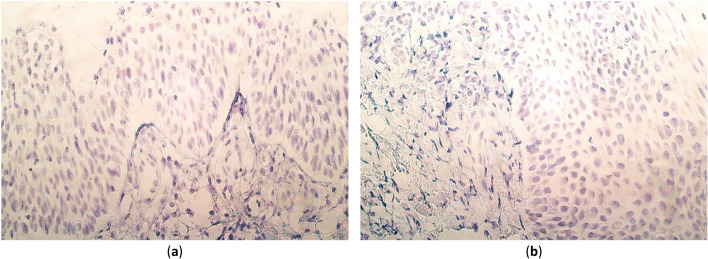
PAX9 and SHH gene visualization in cleft-affected palatine tissue of patients using chromogenic in situ hybridization method and immersion oil. **a** A tissue sample of a 10-month-old child with numerous to abundant (+ +  + / +  +  + +) gene-signal-containing cells in the epithelium, a few to moderate (+ / + +) gene signals in the connective tissue, and no (0) signals in the endothelium. PAX9 CISH, × 400. **b** A tissue sample of a 15-month-old child with a moderate number (+ +) of gene signals in the epithelium, occasional (0/ +) gene signals in the connective tissue, and no (0) gene signals in the endothelium. SHH CISH, × 400

The majority of cases (10 out of 15) contained PAX9 gene signals in the tissue epithelium, ranging from no gene signals (0) to moderate to numerous (+ + / +  + +). However, no gene signals (0) were observed in the connective tissue and endothelium of palatine mucosa (Table 2, Fig. 2a).

Similarly to PAX9, SHH gene signals were observed only in the epithelium, also ranging from no gene signals (0) to moderate to numerous (+ + / +  + +). Although, the majority of cases (9 out of 15) contained no (0) gene signals. Additionally, only one case had moderate to numerous (+ + / +  + +) SHH gene signals in the epithelium, compared to PAX9, which had 5 cases (Table 2, Fig. 2b).

**Fig. 3:**
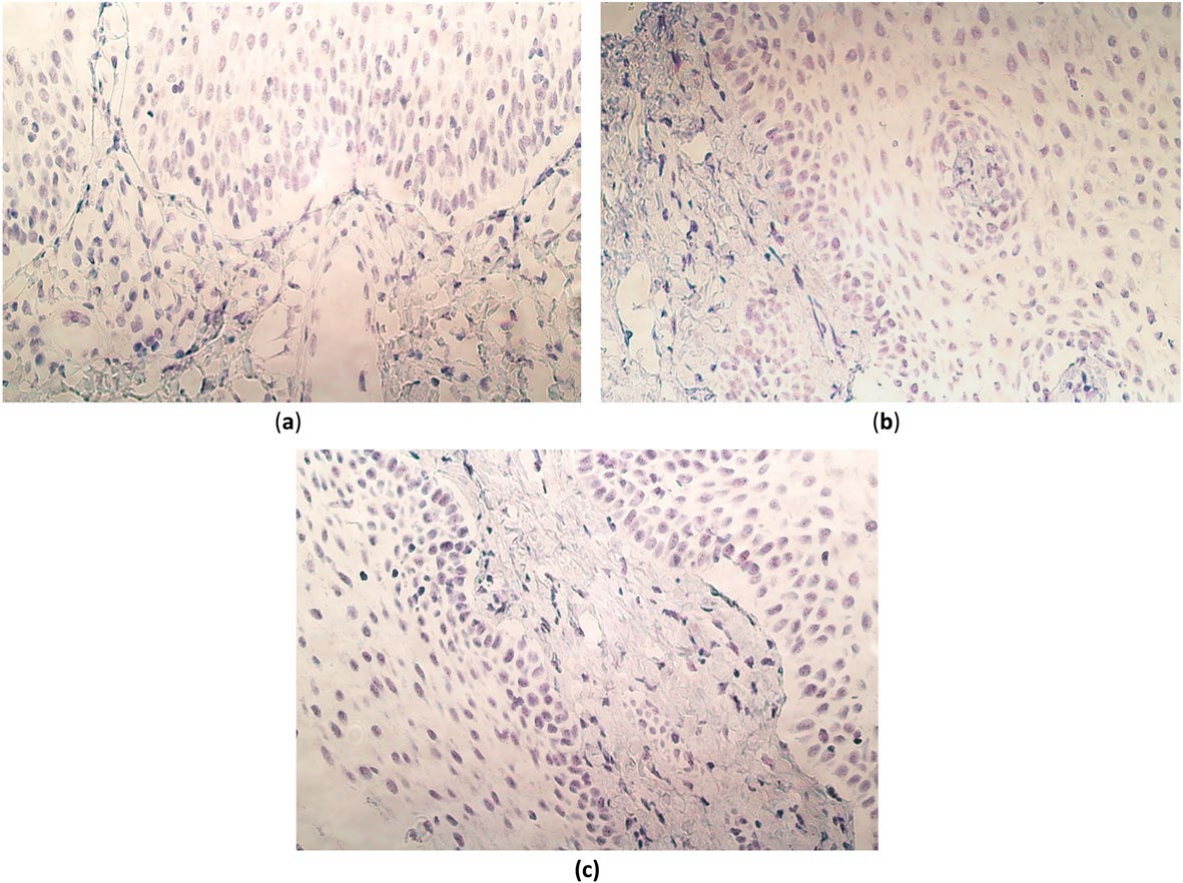
SOX3, WNT3A and WNT9B gene visualization in cleft-affected palatine tissue of patients using chromogenic in situ hybridization method and immersion oil. **a** A tissue sample of a 10-month-old child with a moderate (+ +) number of gene signals in the epithelium, occasional (0/ +) gene signals in the connective tissue, and no (0) gene signals in the endothelium. SOX3 CISH, × 400. **b** A tissue sample of a 15-month-old child with numerous (+ + +) gene signals in the epithelium, occasional (0/ +) gene signals in the connective tissue, and no (0) gene signals in the endothelium. WNT3A CISH, × 400. **c** A tissue sample of a 15-month-old child with numerous to abundant (+ +  + / +  +  + +) gene-signal-containing cells in the epithelium, a few ( +) gene signals in the connective tissue, and no (0) gene-signal-containing cells in the endothelium. WNT9B CISH, × 400

In the epithelium, SOX3 gene signals were observed in the majority of cases (9 out of 15), ranging from no (0) gene signals to numerous (+ + +) (Table 3, Fig. 3a). Although, only one case had numerous SOX3 gene signals in the epithelium. Overall, the median value was 0.5 (Q1–Q3, 0.0–2.0). Interestingly, in the connective tissue and endothelium, SOX3 gene signals were detected only in one case (patient no. 12).

Even though WNT3A and WNT9B gene signals were abundantly observed in the epithelium, similarly to PAX9 and SHH, no (0) gene signals were detected in the connective tissue nor the epithelium (Tables 2 and 3). The range of WNT3A and WNT9B gene signals is the same as PAX7 (from no (0) to numerous to abundant (+ +  + / +  +  + +) gene signals within the palatine mucosa) (Fig. 3b, c). However, whilst the median value of PAX7 was 2.5 (Q1–Q3, 0.5–3.5) in the epithelium, the median value of WNT3A and WNT9B was 0.5 (Q1–Q3, 0.5–2.0) and 0.5 (Q1–Q3, 0.0–3.0), respectively.

In this research, a statistically significant difference in the distribution of PAX7, PAX9, WNT3A, and WNT9B in the epithelium of cleft-affected palatine tissue between the study group and control group was observed (Table 4). Overall, the *p* value varied greatly from 0.005 to > 0.999.

**Table 4:** Statistical importance of the distribution of evaluated genes’ signals between the study and control group

	PAX7			PAX9			SHH		
	*E*	*CT*	*END*	*E*	*CT*	*END*	*E*	*CT*	*END*
Mann–Whitney *U*	7.5	15	30	12.5	37.5	37.5	22.5	37.5	37.5
*p*-value	0.005	0.053	0.553	0.025	> 0.999	> 0.999	0.197	> 0.999	> 0.999
	SOX3			WNT3A			WNT9B		
	*E*	*CT*	*END*	*E*	*CT*	*END*	*E*	*CT*	*END*
Mann–Whitney *U*	15	35	35	7.5	37.5	37.5	12.5	37.5	37.5
*p*-value	0.053	0.866	0.866	0.005	> 0.999	> 0.999	0.025	> 0.999	> 0.999

*Abbreviations*: *E* epithelium, *CT* connective tissue, *END* endothelium

**Fig. 4:**
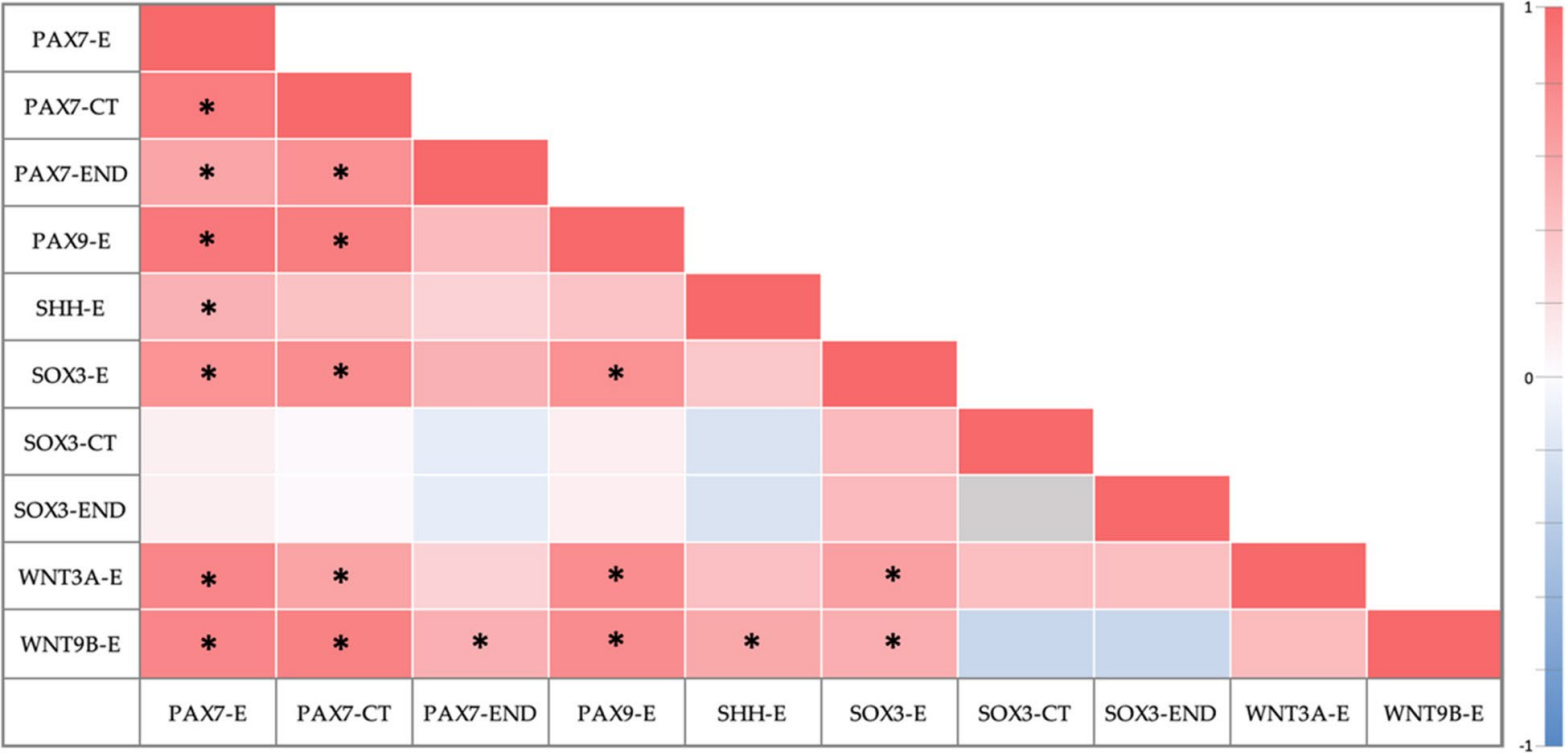
Correlations between genes in palatine tissue samples based on Spearman’s correlation analyses. Note: asterisk (*) within a cell indicates a statistically significant correlation. Grey-colored cell denotes not applicable data. Abbreviations: E-epithelium, CT- connective tissue, END- endothelium

In this research, a statistically significant correlation was observed between 19 pairs (Fig. 4, Supplementary file, Table S2). For some genes, due to the values being “zero” (Tables 2 and 3), no correlation was calculated by Spearman’s test. This data was labeled as “Not applicable” and partially excluded from Fig. 4. However, the complete heatmap and table with exact values of correlations can be viewed in the Supplementary file, Figure S2 and Table S2, respectively.

Overall, a very strong correlation was observed between PAX7 in the epithelium and PAX7 in the connective tissue and PAX9 in the epithelium (R 0.874, *p* < 0.001 and *R* 0.898, *p* < 0.001, respectively). Additionally, PAX9 in the epithelium very strongly correlated with PAX7 in the connective tissue (*R* 0.861, *p* < 0.001).

A very strong association was found between WNT3A in the epithelium and PAX7 in the epithelium (*R* 0.803, *p* < 0.001), however, a strong correlation was observed with PAX7 in the connective tissue (*R* 0.604, *p* = 0.017) and PAX9 and SOX3 in the epithelium (*R* 0.757, *p* = 0.001 and R 0.628, *p* = 0.012, respectively).

Additionally, WNT9B in epithelium very strongly correlated with PAX7 in the connective tissue (*R* 0.836, *p* < 0.001), strongly correlated with PAX7 and PAX9 in the epithelium (*R* 0.796, *p* < 0.001 and *R* 0.764, *p* = 0.001, respectively), but moderately with PAX7 in endothelium (*R* 0.522, *p* = 0.046) and SHH and SOX3 in epithelium (*R* 0.570, *p* = 0.027 and *R* 0.535, *p* = 0.040, respectively).

In the epithelium, SOX3 strongly correlated with PAX7 (*R* 0.711, *p* = 0.003) and PAX9 (*R* 0.727, *p* = 0.002). Also, SOX3 in epithelium strongly correlated with PAX7 in the connective tissue (*R* 0.760, *p* = 0.001).

PAX7 in endothelium strongly correlated with PAX7 in the connective tissue (*R* 0.729, *p* = 0.002), but moderately with PAX7 in the epithelium (*R* 0.590, *p* = 0.021). Lastly, a moderate correlation was observed in the epithelium between PAX7 and SHH (*R* 0.516, *p* = 0.049).

## Discussion

The correct development of orofacial structures is an intricate cascade of events consisting of coordinated cell growth, migration, differentiation, and apoptosis (for review, see Hammond et Dixon [51]). An error in any of the stages could result in an orofacial cleft or other facial abnormality. Orofacial clefts are thought to be caused by a combination of genetic and environmental risk factors. Furthermore, gene-environmental and gene–gene interaction could also play a role in oral cleft etiology [52, 53].

In the present study, to visualize and evaluate gene signals, we used CISH. It is mainly applied in oncology but is high in specificity (~ 94–100%) and sensitivity (~ 94.4–100%) [54]. Compared to fluorescence in situ hybridization, permanent staining, visualization of the results using a standard light microscope, and concurrent observation of gene signals and tissue morphology are some of the benefits of this method [48, 54]. Overall, in our research, the epithelium contained the most gene-signal-containing cells (Tables 2 and 3). Whilst only PAX7 and SOX3 were detected in the connective tissue and endothelium, all genes were observed in the epithelium. In our study group, PAX7 had the highest presence within the tissue samples. Comparing the gene distribution between the study group and control, only PAX7, PAX9, WNT3A, and WNT9B in the epithelium had a statistically important difference (Table 4). Spearman’s rank correlation coefficient revealed 19 pairs of statistically significant, positive correlations (Fig. 4, Supplementary file, Table S2).

Our data revealed that, in comparison with other examined genes, PAX7 had the highest presence in the epithelium and connective tissue of cleft-affected palatine tissue. Therefore, potentially indicating a more significant role of PAX7 in our research group. Studies have shown that PAX7 plays an essential role in neural crest cell development and, in case of impaired function, could lead to orofacial clefts [15, 55, 56]. Whilst we observed varying amounts of PAX7 gene-signal-containing cells throughout the tissue samples in the study group, only in the epithelium is the difference in the distribution statistically significant. Thus, our results are partially in line with another research, where authors observed statistically important differences in PAX7 distribution in the epithelium and connective tissue of unilateral cleft lip in immunohistochemically stained tissue [23]. In mice models, impaired Pax7 function has been reported to result in facial morphogenesis defects [13, 15]. In a study conducted by Mansouri et al., Pax7 − / − mice exhibited defects in facial structures involving the maxilla and nose possibly due to cephalic neural crest defect [15]. However, Zalc et al. noted in mice Pax3/7 is essential in late craniofacial development to control the maintenance of cycling cranial neural crest cell population [13]. Moreover, studies have demonstrated that common and rare PAX7 polymorphisms are associated with an increased risk of non-syndromic CL/P [19, 20]. Taking into account the aforementioned, we believe PAX7 could play a significant role in cleft development, particularly the palatine cleft, in our study group.

In mice models, impaired Pax9 function has been reported to result in an orofacial cleft [16–18]. For example, Zhou et al. observed that Pax9 is essential in the patterning of the anterior–posterior axis and outgrowth of the developing palatal shelves [16]. Moreover, PAX9 polymorphisms have been associated with increased orofacial cleft risk in the Asian population [21, 22]. Overall, research shows that during palatogenesis PAX9 participates in epithelial-mesenchymal communications of palate growth (initiates outgrowth and growth after elevation), palatal shelf elevation, and fusion [14]. In our research, no PAX9 gene-containing cells were observed in the connective tissue or the endothelium. Although the epithelium had varying amounts of PAX9-containing cells, the distribution between the study group and control was statistically significant. In an immunohistochemistry study, the results differed depending on the orofacial cleft type and, for instance, a statistically important difference only in the epithelium was noted between bilateral cleft lip and control [23]. Interestingly, the control group also expressed varying amounts of PAX7 and PAX9 [23]. Conversely, our control group revealed no gene expression in any of the 3 locations examined. We believe there could be a few potential explanations. Overall, it is well known that mRNA and protein expression poorly correlate [57, 58]. In addition, whilst our tissue samples were taken from the soft palatine tissue, Vaivads et al. used oral cavity tissue samples from patients undergoing labial frenectomy due to hypertrophic upper lip frenulum. Lastly, each child’s individual gene and protein expression patterns could have been an influencing factor. In our opinion, PAX9 role in the palatine epithelium and CLP development cannot be excluded.

WNT signaling is essential for directing cell proliferation and polarity, as well as cell fate determination during development and tissue homeostasis [40]. WNT3A seems to play a role in neural crest cell differentiation and migration and regulation of the epithelial-mesenchymal transition [47]. On the other hand, research indicates that during midfacial development Wnt9b might activate the canonical Wnt signaling pathway and possibly accomplish it through the Fzd1 and Fzd2 receptors [59, 60]. Conditional loss or gain of function of β-catenin in palatine epithelium results in a cleft palate and aberrant fusion between the palate shelf and mandible [61]. For instance, in a study by Jin et al., loss of the Wnt9b gene in mice caused retarded outgrowth and failed contact of the nasal and maxillary processes due to reduced proliferation of mesenchymal cells, which in turn disrupted the physical contact between the facial processes that lead to CL/P [42]. Furthermore, different mice and human studies have noted a strong association between OFC and WNT3A and WNT9B [42–47]. In our study, the amount of WNT3A and WNT9B gene-signal-containing cells in the epithelium varied greatly and the distribution differed statistically significantly from the control group. However, no gene-signal-containing cells were observed in the connective tissue nor endotheliocytes in either the study group or the control group. Therefore, potentially indicates a disruption in the local mechanisms, particularly in the epithelium. Nonetheless, the causative role of WNT3A and WNT9B, especially in the palatine epithelium, also cannot be excluded.

SHH, which regulates many aspects of embryonic development, is one of the most important genes with various functions [62]. It is recognized as an important early regulator in reciprocal epithelial-mesenchymal communications controlling epithelial and mesenchymal proliferation [14]. Furthermore, Shh signaling seems to promote pericyte-like function in cranial neural crest cells, which is necessary for microvascular stability and correct facial morphogenesis [63]. SHH has been associated with orofacial clefts in mice and human studies [26–30]. In our research, only 6 tissue samples had varying amounts of SHH gene-containing cells in the epithelium, but the results were not statistically significant. On the other hand, SOX3 is necessary for the formation of the hypothalamic-pituitary axis, and its expression is detected throughout the developing CNS [33]. In mice and humans, abnormal function leads to a variable phenotype. In the case of SOX3 mutations in humans, craniofacial and dental abnormalities, such as solitary median maxillary incisor, high and/or narrow palate, and palatal ridge in the midline, have been reported [37–39]. In our study, the epithelium had the most SOX3-containing cells, but only in one case, we note this gene in all 3 locations. Nonetheless, the results were statistically insignificant. Overall, there is a very limited amount of studies observing an association between SOX3 and orofacial clefts. For example, one study reported increased SOX3-positive immunoreactive structures within human unilateral cleft lip [30]. Therefore, while our data indicates that neither SHH nor SOX3 played a role in our study group, we cannot categorically exclude their role from CLP morhopathogenesis.

In total, we observed 19 pairs of positive correlations which varied from moderate to very strong correlations. Overall, several different pathways, such as Bmp, Tgfβ, Wnt, Shh, Fgf, and their components, are involved in correct orofacial development [64]. Furthermore, these morphogenetic signaling pathways do not act in isolation but interact with one another [61]. Thus, in orofacial cleft tissue, changes in more than one gene are possible, and potential ways of interaction have been described. For instance, Pax7 is thought to be a repressor of Wnt signaling and acts antagonistically to Barx2 in regulating Wnt signaling. However, Pax9 seems to be a downstream effector of Wnt signaling in the palate mesenchyme, as well as can act as an interface between Wnt, Fgf, and Bmp signaling pathways [61]. Jia et al. results of the chromatin immunoprecipitation-polymerase chain reaction (ChIP-PCR) assay revealed that Pax9 is able to bind to the intergenic region of Wnt9b and Wnt3 ligands which are downregulated in Pax9 − / − mice palates [65]. It has been observed that increased Shh signaling indirectly restricts canonical Wnt signaling in the lambdoidal region. Furthermore, reduced canonical Wnt signaling compromised p63/IRF6 activity, resulting in higher proliferation and decreased cell death, which in turn caused the persistence of the epithelial seam and cleft lip development [66]. Studies with mice have revealed that Pax9 regulates a network involving Bmp and Fgf signaling targets which converge on Shh and Osr2 signalling. Whilst in the palatine mesenchyme Pax9 is expressed in a posterior to anterior gradient, Pax9 − / − mice have shown cleft palate with defects in palatal growth/elevation [51]. Zhou et al. reported Pax9 − / − mutants with reduced Shh expression in the palatal epithelium, resulting in abnormal shelf elongation and disorganized rugae morphology [16]. Similarly, Lin et al. noted the loss of Wnt signaling in the palatine epithelium blocked the development of palatal rugae and altered Shh expression. Consequently, a retarded anteroposterior extension of the hard palate formed [67]. Li et al. results showed that the Wnt β-catenin pathway is positively regulated by Pax9, particularly in the middle and posterior regions. Pharmacological inhibition of DKK function in Pax9del/del partly rescued secondary palate development, resulting in a secondary palate fused in the middle and posterior regions [68]. Although a malformed tongue may not be critical for shelf elevation, it is one of the hypotheses for delayed or failed shelf elevation in mice models. Studies have noted that loss of Pax9 expression contributed to later (~ 1 embryonic day) anterior and posterior palatine shelf elevation in mice [14]. Shh and Wnt signaling has also been shown to be essential for tongue formation [67]. Furthermore, Wnt signaling has been reported to act upstream of Notch signaling to maintain the proliferation of the muscle progenitor cells by Pax7. However, in turn, Notch seems to negatively regulate Wnt signaling for normal tongue development [69]. Taking into account the aforementioned information, in our opinion, the indirect correlations in this study represent the complicating interactions of the pathways and their components in the orofacial cleft morphopathogenesis mentioned in the literature.

Interestingly, 2 patients had no gene-signal-containing cells in the palatine tissue, possibly indicating the role of other causative genes which were not examined in this research. On the other hand, the epithelium of one patient had a small amount of only SOX3-containing cells; however, 5 patients had all 6 genes, but the remaining patients had 3–5 different gene signals within their epithelium. In our opinion, in case of multiple gene presence within the tissue, it could indicate a broader disruption in the genetic mechanisms of postnatal orofacial, particularly palate, cleft. Overall, it could indicate a highly individual involvement of all six examined genes in CLP-affected palatine tissue, suggesting a possible difference in postnatal cleft morphopathogenetic mechanisms. We conclude that in our study group, multiple gene involvement mainly affecting the epithelium and PAX7 potentially playing the most important role may have led to OFC development with further postnatal expression into the cleft-affected postnatal tissue.

Due to the difficulties of obtaining this unique palatine tissue material, the main limitation of our study is the small size of the study and the control group. Consequently, more nuanced patterns and intercorrelations could have been missed, and it may interfere with the general applicability of our findings. To our knowledge, there is a limited amount of morphological research examining these genes in human CLP tissue samples; ergo, for instance, extended comparison of results was not possible. Furthermore, correlations between the genes were calculated using statistical methods; hence, we cannot judge whether these genes directly affect one another. Moving further, for future studies, the usage of ChIP assay or similar methods would be necessary.

To conclude, our data seems to support the role of PAX7, PAX9, WNT3A, and WNT9B in CLP development. However, whilst our results indicate no role for SHH and SOX3 in our study group, overall, we cannot exclude their involvement in orofacial cleft morphopathogenesis. Nonetheless, although currently, our observations are yet to have any direct impact on the cleft lip with cleft palate prevention and patient management, discovering all of the causal genes and their potential gene–gene and gene-environmental interactions are relevant to improve overall understanding of orofacial cleft etiology and pathogenesis which in turn might aid to develop new preventative strategies, therapeutic approaches as well as help with genetic counseling.

## Conclusions

Changes in the postnatal CLP-affected palatine epithelium primarily seem to be influenced by the PAX7 gene, whilst PAX9, WNT3A, WNT9B, and SOX3 roles is more limited.

Even though connective tissue changes seem to depend on PAX7 only, SHH seems to participate individually and indistinctly.

The various positive correlations, which varied from moderate to very strong, reflect the complicating interactions of the gene-regulated pathways and their components in postnatal orofacial cleft morphopathogenesis.

### Supplementary Information

**Additional file 1: Table S1.** Characterization of the study group. **Figure S1.** Visual representation of control tissue samples. **Figure S1a.** Control tissue sample without any gene signals in the epithelial cells, connective tissue or endothelium. PAX9 CISH, 1000x. **Figure S1b.** Control tissue sample without any gene signals in the epithelial cells, connective tissue or endothelium. SHH CISH, 1000x. **Figure S1c.** Control tissue sample without any gene signals in the epithelial cells, connective tissue or endothelium. SOX3 CISH, 1000x. **Figure S1d.** Control tissue sample without any gene signals in the epithelial cells, connective tissue or endothelium. WNT3A CISH, 1000x. **Figure S1e.** Control tissue sample without any gene signals in the epithelial cells, connective tissue or endothelium. WNT9B CISH, 1000x. **Table S2.** Exact values of correlations between examined genes in palatine tissue samples based on Spearman’s test. **Figure S2.** Correlations between genes in palatine tissue samples based on Spearman’s correlation analyses.

## Data Availability

The data used and/or analyzed in this study are presented in the results section of the present research and attached supplementary file.

## Abbreviations

Barx2: BARX homeobox 2
Bmp: Bone morphogenetic protein
ChIP: Chromatin immunoprecipitation
CISH: Chromogenic in situ hybridization
CL/P: Cleft lip with or without a cleft palate
CLO: Cleft lip only
CLP: Cleft lip with cleft palate
CNS: Central nervous system
CPO: Isolated cleft palate
CT: Connective tissue
DKK: Dickkopf
DNA: Deoxyribonucleic acid
E: Epithelium
END: Endothelium
Fgf: Fibroblast growth factor
Fzd1: Frizzled class receptor 1
Fzd2: Frizzled class receptor 2
IRF6: Interferon regulatory factor 6
OFC: Orofacial cleft
Osr2: Odd-skipped related transcription factor 2
p63: Tumor protein P63
Pax3: Paired box gene 3
PAX7: Paired box gene 7
PAX9: Paired box gene 9
PCR: Polymerase chain reaction
RNA: Ribonucleic acid
SHH: Sonic hedgehog
SOX: SRY-related HMG-box
SOX1: SRY-related HMG-box gene 1
SOX2: SRY-related HMG-box gene 2
SOX3: SRY-related HMG-box gene 3
SUDC: Sudden unexplained death in childhood
Tgfβ: Transforming growth factor beta
WNT: Wingless-type MMTV integration site
WNT3A: Wingless-type MMTV integration site family, member 3A
WNT9B: Wingless-type MMTV integration site family, member 9B
