# Supplementary material for: The presence and distribution of various genes in postnatal CLP-affected palatine tissue
Source: Maxillofac Plast Reconstr Surg. 2024 Jan 16;46(1):1. doi: 10.1186/s40902-024-00412-1 (PMC10792134; doi:10.1186/s40902-024-00412-1)
Supplement: Supplementary file 1 — Additional file 1: Table S1. Characterization of the study group. Figure S1. Visual representation of control tissue samples. Figure S1a. Control tissue sample without any gene signals in the epithelial cells, connective tissue or endothelium. PAX9 CISH, 1000x. Figure S1b. Control tissue sample without any gene signals in the epithelial cells, connective tissue or endothelium. SHH CISH, 1000x. Figure S1c. Control tissue sample without any gene signals in the epithelial cells, connective tissue or endothelium. SOX3 CISH, 1000x. Figure S1d. Control tissue sample without any gene signals in the epithelial cells, connective tissue or endothelium. WNT3A CISH, 1000x. Figure S1e. Control tissue sample without any gene signals in the epithelial cells, connective tissue or endothelium. WNT9B CISH, 1000x. Table S2. Exact values of correlations between examined genes in palatine tissue samples based on Spearman’s test. Figure S2. Correlations between genes in palatine tissue samples based on Spearman’s correlation analyses. [file 40902_2024_412_MOESM1_ESM.pdf]

# Supplementary file

**Table S1.** Characterization of the study group.

| No. | Gender | Diagnosis (Latin) | Diagnosis (English) | Age in months | Notes |
|---|---|---|---|---|---|
| 1. | Male | Cheilognathouranoschisis sinistra | Left unilateral cleft lip, alveolus and palate | 4 | Paracetamol had been used during pregnancy. |
| 2. | Male | Cheilognathouranoschisis sinistra | Left unilateral cleft lip, alveolus and palate | 8 | Mother with a cleft lip and palate. |
| 3. | Female | Cheilognathouranoschisis sinistra | Left unilateral cleft lip, alveolus and palate | 9 | |
| 4. | Male | Cheilognathouranoschisis dextra | Right unilateral cleft lip, alveolus and palate | 9 | |
| 5. | Female | Cheilognathouranoschisis sinistra | Left unilateral cleft lip, alveolus and palate | 9 | |
| 6. | Female | Cheilognathouranoschisis dextra | Right unilateral cleft lip, alveolus and palate | 9 | |
| 7. | Male | Cheilognathouranoschisis bilateralis | Bilateral cleft lip, alveolus and palate | 9 | |
| 8. | Male | Cheilognathouranoschisis sinistra | Left unilateral cleft lip, alveolus and palate | 10 | |
| 9. | Male | Cheilognathouranoschisis dextra | Right unilateral cleft lip, alveolus and palate | 10 | |
| 10. | Male | Cheilognathouranoschisis sinistra | Left unilateral cleft lip, alveolus and palate | 10 | Down syndrome present in family history. |
| 11. | Male | Cheilognathouranoschisis bilateralis | Bilateral cleft lip, alveolus and palate | 10 | |
| 12. | Female | Cheilognathouranoschisis sinistra | Left unilateral cleft lip, alveolus and palate | 11 | |
| 13. | Male | Cheilognathouranoschisis sinistra | Left unilateral cleft lip, alveolus and palate | 14 | |
| 14. | Male | Cheilognathouranoschisis sinistra | Left unilateral cleft lip, alveolus and palate | 15 | Paracetamol had been used during pregnancy. |
| 15. | Female | Cheilognathouranoschisis dextra | Right unilateral cleft lip, alveolus and palate | 48 | |

*Abbreviations: No.- patient's number*

**Figure S1.** Visual representation of control tissue samples.

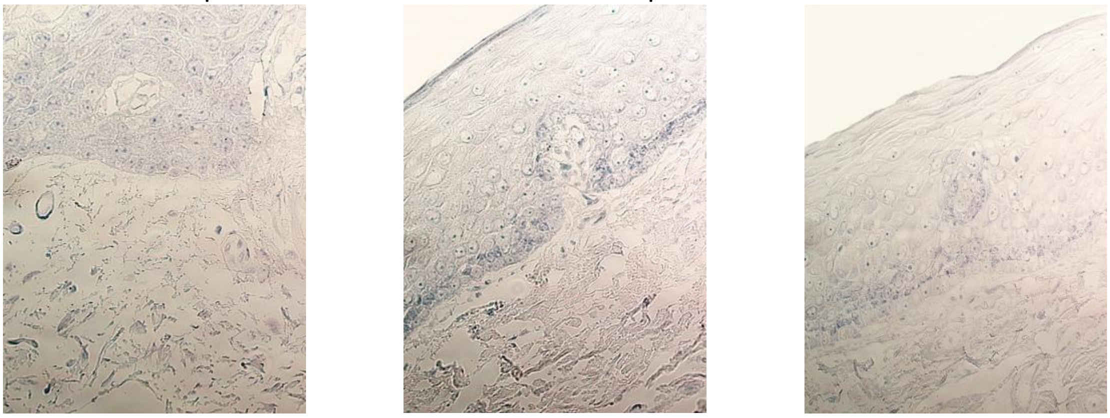

(a) (b) (c)

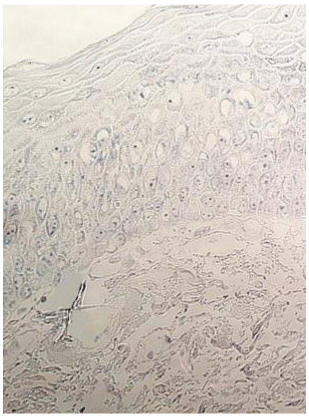

(d)

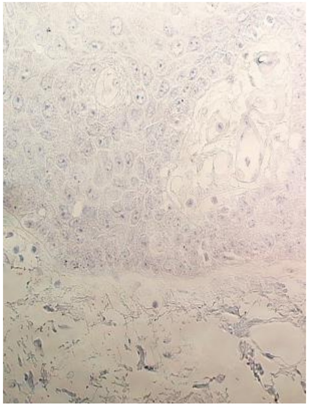

(e)

**Figure S1a**. Control tissue sample without any gene signals in the epithelial cells, connective tissue or endothelium. PAX9 CISH, 1000x.
**Figure S1b**. Control tissue sample without any gene signals in the epithelial cells, connective tissue or endothelium. SHH CISH, 1000x.
**Figure S1c**. Control tissue sample without any gene signals in the epithelial cells, connective tissue or endothelium. SOX3 CISH, 1000x.
**Figure S1d**. Control tissue sample without any gene signals in the epithelial cells, connective tissue or endothelium. WNT3A CISH, 1000x.
**Figure S1e**. Control tissue sample without any gene signals in the epithelial cells, connective tissue or endothelium. WNT9B CISH, 1000x.

**Table S2**. Exact values of correlations between examined genes in palatine tissue samples based on Spearman's test.

| Factor 1 | Factor 2 | R | *p*-value |
|---|---|---|---|
| **Very strong correlation** | | | |
| PAX7-E | PAX7-CT | 0.874 | 0.000 |
| PAX7-E | PAX9-E | 0.898 | 0.000 |
| PAX7-E | WNT3A-E | 0.803 | 0.000 |
| PAX7-CT | PAX9-E | 0.861 | 0.000 |
| PAX7-CT | WNT9B-E | 0.836 | 0.000 |
| **Strong correlation** | | | |
| PAX7-E | SOX3-E | 0.711 | 0.003 |
| PAX7-E | WNT9B-E | 0.796 | 0.000 |
| PAX7-CT | PAX7-END | 0.729 | 0.002 |
| PAX7-CT | SOX3-E | 0.760 | 0.001 |
| PAX7-CT | WNT3A-E | 0.604 | 0.017 |
| PAX9-E | SOX3-E | 0.727 | 0.002 |
| PAX9-E | WNT3A-E | 0.757 | 0.001 |
| PAX9-E | WNT9B-E | 0.764 | 0.001 |
| SOX3-E | WNT3A-E | 0.628 | 0.012 |

| Factor 1 | Factor 2 | R | *p*-value |
|---|---|---|---|
| **Moderate correlation** | | | |
| PAX7-E | PAX7-END | 0.590 | 0.021 |
| PAX7-E | SHH-E | 0.516 | 0.049 |
| PAX7-CT | SHH-E | 0.400 | 0.140 |
| PAX7-END | PAX9-E | 0.451 | 0.092 |
| PAX7-END | SOX3-E | 0.513 | 0.051 |
| PAX7-END | WNT9B-E | 0.522 | 0.046 |
| SHH-E | WNT3A-E | 0.411 | 0.128 |
| SHH-E | WNT9B-E | 0.570 | 0.027 |
| SOX3-E | SOX3-CT | 0.450 | 0.093 |
| SOX3-E | SOX3-END | 0.450 | 0.093 |
| SOX3-E | WNT9B-E | 0.535 | 0.040 |
| SOX3-CT | WNT3A-E | 0.418 | 0.121 |
| SOX3-END | WNT3A-E | 0.418 | 0.121 |
| WNT3A-E | WNT9B-E | 0.440 | 0.101 |

| Factor 1 | Factor 2 | R | *p*-value |
|---|---|---|---|
| **Weak correlation** | | | |
| PAX7-END | SHH-E | 0.281 | 0.309 |
| PAX7-END | WNT3A-E | 0.283 | 0.306 |
| PAX9-E | SHH-E | 0.393 | 0.147 |
| SHH-E | SOX3-E | 0.363 | 0.184 |
| SHH-E | SOX3-CT | -0.211 | 0.451 |
| SHH-E | SOX3-END | -0.211 | 0.451 |
| SOX3-CT | WNT9B-E | -0.317 | 0.249 |
| SOX3-END | WNT9B-E | -0.317 | 0.249 |
| **Very weak correlation** | | | |
| PAX7-E | SOX3-CT | 0.095 | 0.736 |
| PAX7-E | SOX3-END | 0.095 | 0.736 |
| PAX7-CT | SOX3-CT | 0.032 | 0.909 |
| PAX7-CT | SOX3-END | 0.032 | 0.909 |
| PAX7-END | SOX3-CT | -0.133 | 0.637 |
| PAX7-END | SOX3-END | -0.133 | 0.637 |
| PAX9-E | SOX3-CT | 0.097 | 0.732 |
| PAX9-E | SOX3-END | 0.097 | 0.732 |

*Abbreviations: E-epithelium, CT- connective tissue, END-endothelium*

**Figure S2**. Correlations between genes in palatine tissue samples based on Spearman's correlation analyses.

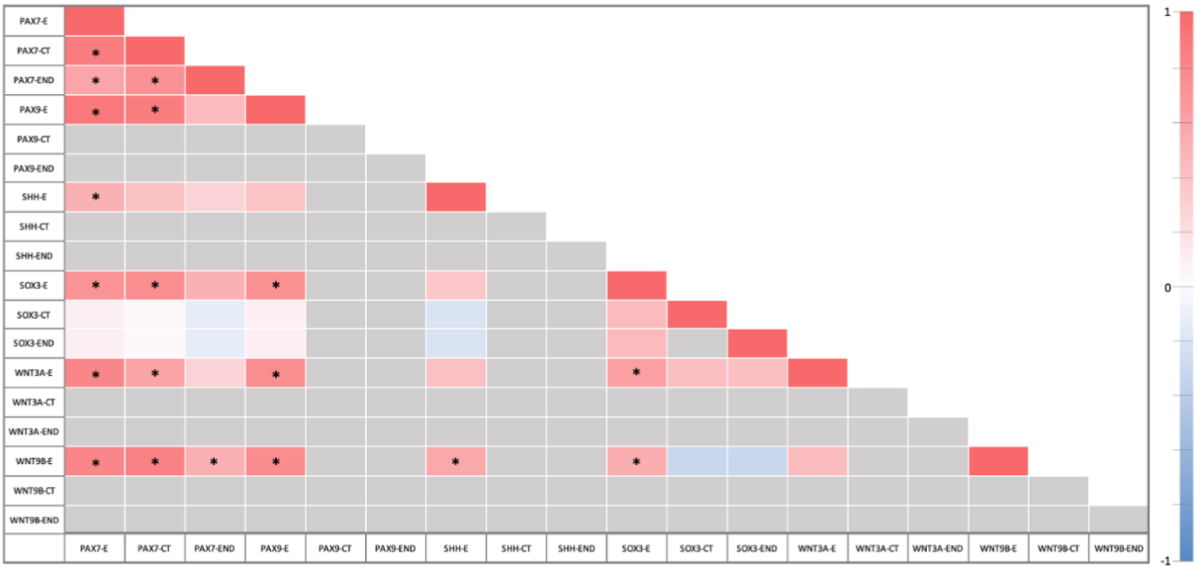


Note: asterisk (*) within a cell indicates a statistically significant correlation. Grey-coloured cells denote not applicable data.

*Abbreviations: E-epithelium, CT- connective tissue, END- endothelium*.
